# Selection of surgical modality for massive splenomegaly in children

**DOI:** 10.1007/s00464-023-10462-7

**Published:** 2023-10-05

**Authors:** Yong Li, Congjun Wang, Weilong Chen, Chao Chen, Xianming Tang, Hong Wang, Jiabo Chen, Qiang Liu, Wei Li, Yanqiang Li, Peng Chen, Yige Luo, Cheng Su

**Affiliations:** 1 Department of Pediatric Surgery, The First Affiliated Hospital of Guangxi Medical University, Shuangyong Road No.6, Region Qingxiu, Nanning, 530021 Guangxi Province China; 2 Department of Pediatric Surgery, Zhuhai Women and Children’s Hospital, Ningxi Road No.543, Region Xiangzhou, Zhuhai, 519000 Guangdong Province China

**Keywords:** Splenomegaly, Surgical modality, Surgical outcome, Laparoscopy, Paediatric surgery

## Abstract

**Background:**

Laparoscopic splenectomy (LS), a treatment for both benign and malignant splenic diseases, can prove technically challenging in patients with massive splenomegaly. In particular, the optimal surgical modality for treating massive splenomegaly in children remains controversial.

**Methods:**

The clinicopathologic data of 289 pediatric patients undergoing splenectomy for massive splenomegaly were studied in a retrospective analysis. Accordingly, the patients were classified into the LS surgery group and open splenectomy (OS) surgery group. In the laparoscopy cohort, they were separated into two subgroups according to the method of surgery: the multi-incision laparoscopic splenectomy (MILS) and the single-incision laparoscopic splenectomy (SILS) surgery groups, respectively. Patient demographics, clinical data, surgery, complications, and postoperative recovery underwent analysis. Concurrently, we compared the risk of adverse laparoscopic splenectomy outcomes utilizing univariable and multivariable logistic regression.

**Results:**

The total operation time proved remarkably shorter in the OS group in contrast to the LS group (149.87 ± 61.44 versus 188.20 ± 52.51 min, *P* < 0.001). Relative to the OS group, the LS group exhibited lowered postoperative pain scores, bowel recovery time, and postoperative hospitalization time (*P* < 0.001). No remarkable difference existed in post-operation complications or mortality (*P* > 0.05). Nevertheless, the operation duration was remarkably longer in the SILS surgery group than in the MILS surgery group (200 ± 46.11 versus 171.39 ± 40.30 min, *P* = 0.02). Meanwhile, the operative duration of MILS and SILS displayed a remarkable positive association with splenic length. Moreover, the operative duration of SILS displayed a remarkable positive association with the age, weight, and height of the sick children. Splenic length proved an independent risk factor of adverse outcomes (*P* < 0.001, OR 1.378).

**Conclusions:**

For pediatric patients with massive splenomegaly who can tolerate prolonged anesthesia and operative procedures, LS surgery proves the optimal treatment regimen. SILS remains a novel surgery therapy which may be deemed a substitutional surgery approach for treating massive splenomegaly.

Splenomegaly is frequently observed in children with benign and malignant hematologic conditions, including α- and β-thalassemia, hereditary spherocytosis, lymphoma, and sickle-cell anemia [1]. In this regard, splenectomy proved able to effectively alleviate the symptoms and promote recovery [2]. Currently, primarily 2 types of surgical approaches are in use: open splenectomy (OS) and Laparoscopic splenectomy (LS).

Since its introduction in the early 1990s, LS has gained worldwide acceptance for spleen removal. In contrast to the conventional OS, LS has become a normal method for splenectomy due to its relative advantages, including lowered post-operative pain, shorter hospitalization, and improved quality of life [3, 4]. On the other hand, it can prove technically challenging in patients with massive splenomegaly. With the advancement of laparoscopic techniques and instruments, laparoscopy is considered the gold standard for a range of surgical indications. However, the operative space for laparoscopic surgery is limited, requiring a high level of surgical expertise for the operator.

Representative studies have demonstrated [5, 6] that laparoscopic splenectomy surgery is safe and feasible and exhibits better outcomes than open splenectomy surgery. Nevertheless, given an increase in spleen volume, the difficulties and risks of laparoscopy surgery were notably elevated. The elevated risks are attributed to the enlargement of the spleen and the shrinking of the space where a laparoscope can operate, which exposes greater challenges related to the use of this technology, such as the difficulty of manipulating organs, the decreased surgical field of view, and the complexity of extracting larger samples[7, 8]. Nonetheless, other studies have revealed [9] that laparoscopic splenectomy surgery generally results in better outcomes relative to open surgery. The duration of the operation is more extended in the laparoscopic method in contrast to the laparotomic method; nonetheless; patients benefit from fewer adhesions, shorter hospitalization, and more rapid recovery. In this regard, single-incision laparoscopy is a new development in laparoscopy (although it has now been over 20 years since this technology was developed) which aims to generate diminished invasion. At present, this technique is employed in children's splenectomy surgery. Raboei et al. [10] have confirmed that single-incision laparoscopy was feasible, effective, and safe for splenomegaly. Furthermore, while the safety of single-incision laparoscopic surgery in adult has been validated, the experience with this surgical technique is still very limited in children and young adults. Nowadays, whether such a surgical procedure ought to be implemented for children with massive splenomegaly is a matter of substantial debate.

Although several surgical options exist in children's splenectomy surgery, there is a lack of consensus on optimal surgical management. We analyzed the data from our ongoing cohort study on children's splenectomy in our hospital, comparing the perioperative and long-term results of OS, MILS, and SILS in our research center, with the aim of providing a new theoretical basis for splenectomy in children with massive splenomegaly.

## Materials and methods

### Patients

This study reviewed the retrospective data of sufferers undergoing curative complete splenectomy for massive splenomegaly at The First Affiliated Hospital of Guangxi Medical University between July 2013 and December 2020. Massive splenomegaly was defined as a splenic length of ≥ 15 cm, and the ages ranged from 4 years old to 18 years old. In addition, written informed consent was acquired from all children’s parents, and this research was approved by the ethical board of our university’s affiliated hospital.

### Surgeon procedures

All operations were performed by the same surgeon in our hospital; in the provision of neural anesthesia, patients were placed in the supine position with the torso is tilted 30° to the right. First, a 1.5 cm long (approximately) arc-shaped incision was made below the navel, whereas MILS generated 3–5 incisions in the abdomen. The CO2 pneumoperitoneum was established at 8–12 mmHg.

After two or three stitches of suspension suture were made on stomach wall, the stomach was pulled to the upper right side. Thereafter, the left lateral abdominal wall was sutured around the lower pole of the spleen, and the spleen was pulled to expose the splenic hilum. Then, an ultrasound scalpel was used to cut off the spleen-gastric ligament and the short gastric vessels. The splenic artery was dissociated and ligated. The splenic lower pole vessels were clipped and divided after division of the plenocolic ligament, and subsequently, the splenic upper pole vessels were isolated and processed. The spleno-diaphragmatic and splenorenal ligaments were detached using an ultrasound scalpel from top to bottom to dissociate the spleen completely. Thereafter, the spleen was inserted in a specimen bag and removed from the abdominal l cavity, and a drainage tube was placed in the abdomen or pelvic cavity. Finally, the umbilical cord incision was closed.

### Data collection

In the present study, a total of 289 patients were retrospectively assessed through a review of their medical records. 162 patients with massive splenomegaly had been subjected to LS treatment, and 27 sufferers had been subjected to OS treatment. Posterior to analyses of the overall cohort, 15 patients of the LS group were excluded due to the transfer to open operation during surgery. We included 147 patients (114 in the MILS cohort and 33 in the SILS cohort) in the LS and dialysis groups. To control baseline differences, we conducted the propensity-score matching in R, using the "MatchIt" package. Our team performed a 1:1 matching of MILS to SILS based on age, weight, height, BMI, primary disease, and splenic length. Observation indexes involved demographic data, previous medical history, imaging data, operation time, blood loss volume, postoperative pain score, time of bowel function recovery, hospitalization length, and complications.

To explore the effect of LS on adverse outcomes, our team developed a questionnaire to determine the criteria for evaluating adverse outcomes. Each subject enrolled in the survey was a specialist with a professional title of associate professor or above. The studies were grouped and evaluated according to the criteria.

### Statistical analysis

A statistic program (IBM, America) was utilized for analyses. For descriptive statistics, continuous variables were described as mean ± SD (range) or medians, whereas categorical variables were described as frequencies with percentages. Statistical analyses were performed with the student's *t*-test, Fisher exact test, ANOVA, or *χ*^2^-test. Values of *P* < 0.05 were taken as statistically significant.

## Results

### Open surgery versus laparoscopic surgery

**Fig. 1:**
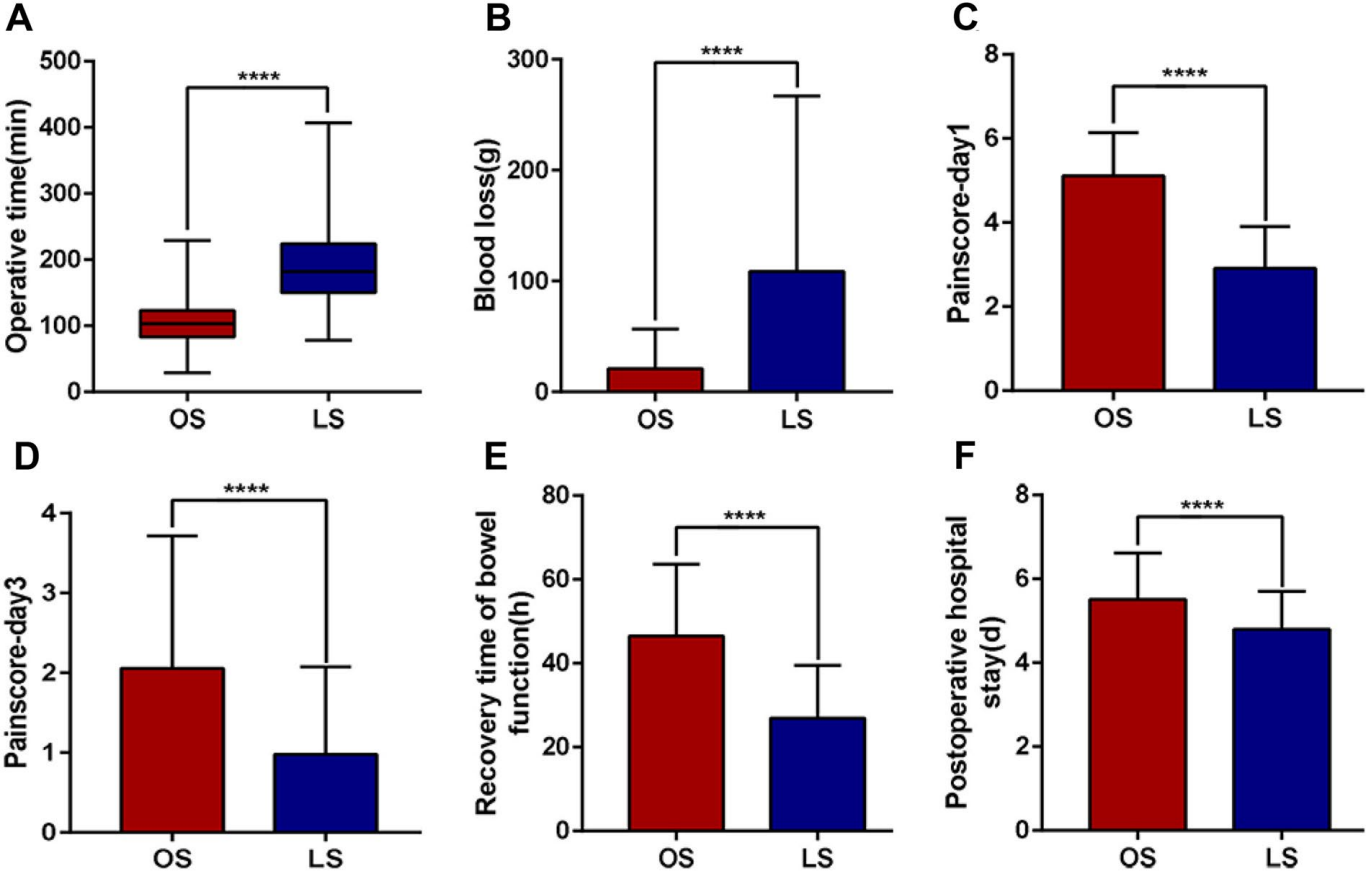
Comparison of the operation time, intraoperative blood loss, pain score, bowel function recovery, and postoperative hospitalization time between the OS and LS groups. **A** Operation time was significantly longer in the LS group (*P* < 0.0001) than in the OS group. **B** Blood loss was significantly lower in the OS group (*P* < 0.0001) than in the LS group. **C**, **D** Pain score in the LS group is significantly lower than in the OS group. **E** The recovery time of bowel function was significantly shorter in the LS group (*P* < 0.0001) than in the OS group. **F** Postoperative hospitalization time was significantly shorter in the LS group (*P* < 0.0001) than in the OS group. In the figure, ****P < 0.0001

To explore the surgical safety of splenectomy in children with massive splenomegaly, a comparison of operative and peri-operative data between the two groups is shown in Table 1 and Fig. 1. Between the two groups, no remarkable differences existed in terms of gender, age, height, weight, underlying disease, and spleen size (*P* > 0.05). However, the operative duration of the LS group was significantly longer in contrast to the OS group, and more intraoperation blood loss by the LS group (*P* < 0.001). All the same, in terms of the postoperative pain scores, bowel recovery time, and postoperative hospitalization duration, the LS group proved better (*P* < 0.001). There was no difference in the incidence of postoperative complications between the two groups (*P* > 0.05).

**Table 1:** Comparison of open versus laparoscopic splenectomy

Variable	OS group (*N* = 127)	LS group (*N* = 147)	*P*-value
Age (years)	8 (5–10)	8 (6–11)	0.213
Gender (M/F, *n*)	76/51	81/66	0.429
Height (cm)	118.30 ± 17.24	121.27 ± 17.40	0.236
Weight (kg)	21.17 ± 3.43	23.29 ± 7.70	0.202
BMI (kg/m^2^)	15.35 ± 1.56	15.52 ± 2.21	0.992
Primary disease			0.767
Thalassemia (*n*, %)	115 (90.6)	130 (88.4)	
Hereditary spherocytosis (*n*, %)	10 (7.9)	13 (8.8)	
Other spleen diseases (*n*, %)	2 (1.5)	4 (2.8)	
Splenic length (cm)	20.5 (19.1–22.6)	20.2 (18.4–22.4)	0.114
Operative time (min)	149.87 ± 61.44	188.20 ± 52.51	< 0.001
Blood loss (g)	10 (10–15)	30 (10–100)	< 0.001
Postoperative pain score			
Day 1	5 (5–6)	3 (2–3)	< 0.001
Day 3	2 (1–3)	1 (0–2)	< 0.001
Bowel function recovery (h)	45 (31–60)	25 (19–33)	< 0.001
Postoperative hospitalization (day)	5 (5–6)	5 (4–5)	< 0.001
Early complications (*n*, %)	14 (11.0)	19 (12.9)	0.630
Late complications (*n*, %)	2 (1.5)	1 (0.7)	0.598
Mortality (*n*, %)	0 (0)	0 (0)	1.000

### SILS surgery versus MILS surgery

To further explore the surgical safety of the SILS group and MILS group, the 147 patients undergoing the laparoscopic surgery were divided into the SILS (*n* = 33) and MILS (*n* = 114) groups. As presented in Table 2, significant differences were exhibited between the two intraoperative preoperative data; after screening 147 patients by propensity-score matching, a total of 56 individuals were included in the study. According to demographic parameters, such as age, sex, weight, height, BMI, spleen size, and primary venereal disease, no differences were exhibited between the two groups (Table 2). We compared the two groups (SILS group and MILS group) regarding surgical characteristics and surgical complications. The operation time in the SILS group was longer than that in the MILS group (200 ± 46.11 min versus 171. 0.30 min, *P* = 0.02). Meanwhile, there was no significant difference in intraoperative blood loss, postoperative pain scores, bowel recovery time, postoperative hospital stay, and postoperative complications between the two groups (Table 3). The two groups showed similar results in terms of the safety and success of the procedure. However, SILS group proved better in the incisional cosmesis than MILS group.

**Table 2:** Baseline characteristics of MILS versus SILS

Variable	Before matching		*P*-value	After matching		*P*-value
	MILS *n* = 114	SILS *n* = 33		MILS *n* = 28	SILS *n* = 28	
Age (years)	8 (5–10)	10 (7–12)	0.002	8 (5.25–9)	10 (6.25–12)	0.068
Gender (M/F, *n*)	59/55	22/11	0.129	18/10	18/10	1.000
Height (cm)	118.61 ± 16.06	130.52 ± 18.83	0.002	121 ± 16.39	128.61 ± 20.56	0.129
Weight (kg)	22.27 ± 6.73	26.83 ± 9.68	0.015	22.88 ± 6.87	25.54 ± 8.11	0.238
BMI (kg/m^2^)	15.60 ± 2.22	15.27 ± 2.16	0.505	15.33 ± 1.45	15.14 ± 1.79	0.762
Primary disease (%)			0.811			0.684
Thalassemia (*n*, %)	99 (86.8)	30 (90.9)		24 (85.8)	26 (92.8)	
Hereditary spherocytosis (*n*, %)	10 (8.8)	2 (6.1)		2 (7.1)	1 (3.6)	
Other spleen diseases (*n*, %)	5 (4.4)	1 (3.0)		2 (7.1)	1 (3.6)	
Splenic length (cm)	19 (18–22)	20 (18–21)	0.449	18 (18–23.5)	20 (18–20.75)	0.380

**Table 3:** The outcome of MILS versus SILS

Variable	MILS *n* = 28	SILS *n* = 28	*P*-value
Operative time (min)	171.39 ± 40.30	200 ± 46.11	0.02
Blood loss (g)	27.5 (11.25–187.5)	20 (10–137.5)	0.525
Postoperative pain score			
Day 1	3 (2–4)	3 (2–3)	0.569
Day 3	1 (0–1)	1 (0–1)	0.607
Bowel function recovery (h)	24.5 (18.75–36.25)	23.5 (19.25–26.75)	0.313
Postoperative hospitalization (day)	5 (4–5)	4 (4–5.75)	0.448
Early complications, *n* (%)	3	0	0.235
Late complications, *n* (%)	0	1	1.000
Mortality, *n* (%)	0	0	1.000

### Clinical factors that affect the total operation time of LS

**Fig. 2:**
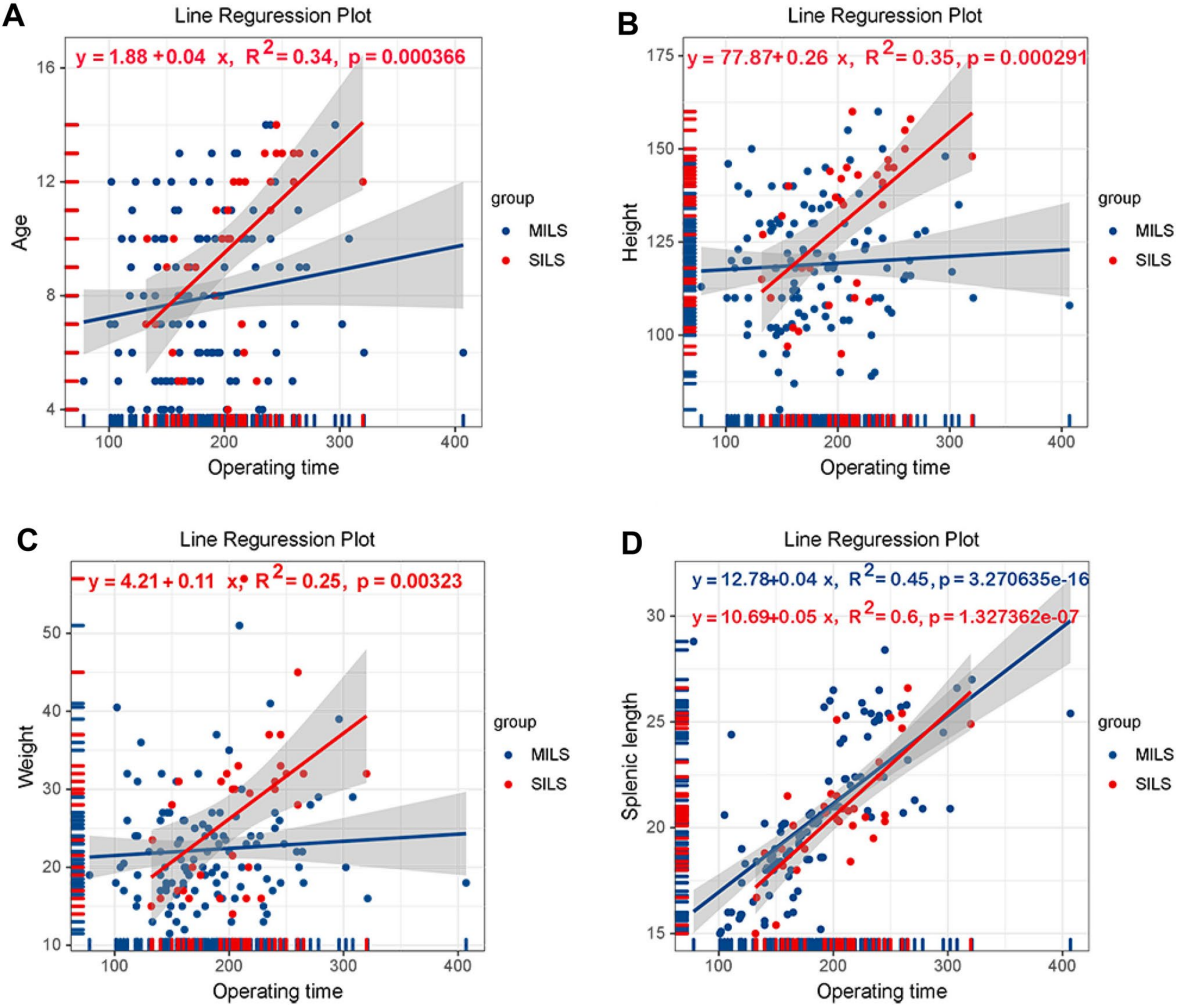
Linear regression analysis of the operative times. **A** Regression analysis of operative time and age in SILS and MILS showed a positive correlation between operative time and age in SILS (*R*^2^ = 0.34). **B** Linear regression revealed a positive correlation between operative time and height in SILS (*R*^2^ = 0.35). **C** Linear regression revealed a positive correlation between operative time and weight in SILS (*R*^2^ = 0.25). **D** Linear regression showed that operative time and splenic length positively correlated with SILS and MILS (SILS: *R*^2^ = 0.6, MILS: *R*^2^ = 0.45)

In order to explore factors pertaining to operation time in LS, a linear regression analysis was performed, using operation time as dependent variable, and age, height, weight, BMI and splenic length as independent variables, respectively in SILS (*n* = 33) and MILS (*n* = 114). In this instance, a significant positive correlation was found between operation times and splenic length in SILS group and MILS group (MILS: *R*^2^ = 0.45, *P* < 0.001, SILS: *R*^2^ = 0.6, *P* < 0.001). In addition, the operation times in SILS showed a significant positive correlation with patient age (*R*^2^ = 0.34, *P* < 0.001), height (*R*^2^ = 0.35, *P* < 0.001) and weight (*R*^2^ = 5, *P* = 0.003). This observation indicated that the splenic length exerts a major influence on the operation time. Table 3 and Fig. 2 illustrate these results.

### The risk factors of LS adverse outcome

In total, 58 returned questionnaires were valid. According to the questionnaire results, the proposed diagnostic criteria for the adverse outcome of LS were intraoperative major bleeding (≥ 400 g), conversion to open surgery, injury of adjacent viscera, postoperative trauma or bleeding (≥ 100 g), postoperative systemic infection, postoperative pancreatic complications, and portal veins thrombosis.

A total of 162 patients were included in this study. We conducted a retrospective cohort analysis. The results are given in Table 5. Accordingly, 34 patients (21.0%) were classified as experiencing an unfavorable outcome, and 128 patients (79.0%), with a favorable outcome. The adverse outcome group exhibited a total of 48 serious adverse events, with 15 patients converted to open surgery, while intraoperative major bleeding was noted in 16 patients; injury of adjacent viscera was reported in two patients; postoperative major bleeding was noted in eight patients; and seven patients experienced postoperative systemic infection, postoperative pancreatic complications, or portal veins thrombosis, as depicted in Table 4. In terms of results from the univariate analysis of clinical data of adverse outcome groups and favorable outcome groups, operation time and splenic length bore significant differences. However, there were no significant differences in age, sex, weight, height, BMI, and surgical mode (Table 5). Accordingly, binary logistic regression analysis was conducted, which showed splenic length (*P* < 0.001, OR 1.378, 95%CI 1.163–1.632) to be an independent risk factor for adverse outcomes in child patients with laparoscopic splenectomy (Table 6).

**Table 4:** Adverse outcome of laparoscopic splenectomy

Serious adverse events of LS	Number of cases, *n*(%)
Convert to open surgery	15 (9.2)
Injury of adjacent viscera	2 (1.2)
Intraoperative major bleeding (≥ 200 g)	16 (9.8)
Postoperative major bleeding (≥ 100 g)	8 (4.9)
Postoperative systemic infection	2 (1.2)
Postoperative pancreatic complications	3 (1.9)
Portal veins thrombosis	2 (1.2)

**Table 5:** Univariate analysis of adverse versus good outcome for LS

Variable	Adverse outcome group (*n* = 34)	Good outcome group (*n* = 128)	*P*-value
Age (years)	10 (6–12)	8 (6–11)	0.188
Gender (M/F, *n*)	18/16	70/58	0.856
Height (cm)	126.91 ± 16.97	124.07 ± 18.22	0.178
Weight (kg)	24.62 ± 6.19	24.20 ± 8.65	0.314
BMI (kg/m^2^)	15.18 ± 1.97	15.52 ± 1.82	0.601
Primary disease (%)			0.666
Thalassemia (*n*, %)	32 (94.12)	115 (89.84)	
Hereditary spherocytosis (*n*, %)	2 (5.88)	11 (8.59)	
Other spleen diseases (*n*, %)	0 (0)	2 (1.57)	
Splenic length (cm)	23.11 ± 3.13	20.02 ± 3.01	< 0.001
Surgical mode			0.811
MILS	25 (73.53)	99 (77.34)	
SILS	9 (26.47)	29 (22.66)	
Operative time	215.50 ± 46.34	183.23 ± 53.01	< 0.001

**Table 6:** Multivariate binary analysis of poor versus good outcome for LS

	*B*	*SE*	Wald	*P*-value	OR	95%CI
Splenic length	0.320	0.086	13.745	< 0.001	1.378	1.163–1.632
Operative time	− 0.001	0.005	0.047	0.829	0.999	0.989–1.009

## Discussion

In the surgical treatment of spleen diseases in adult populations, laparoscopy has been increasingly employed. Numerous studies have indicated [11–14] that laparoscopic splenectomy is associated with significantly less surgical trauma, less postoperative pain, shortened hospitalization, fewer complicating diseases, and a higher quality of life than the conventional open method. As such, this surgical technique is worthy of extensive clinical application. Laparoscopic spleen surgeries can be implemented safely, they require abundant open spleen surgery experience and expertise in laparoscopy operation on the part of the surgeon. Nonetheless, reports on the application of laparoscopic splenectomy techniques in pediatric populations are currently insufficient. In this study, follow-up results suggest that laparoscopic splenectomy is feasible and safe for children with massive splenomegaly. In contrast to the conventional OS approaches, the post-operation pain is less, and the recovery time is more rapid, which also indirectly implies that laparoscopic surgery is less invasive. On the other hand, laparoscopic splenectomy is a more complicated and requires a longer operating time. Although technically challenging, LS surgery may provide a clearer view of the surgical field than open surgery, which may facilitate the resection of the spleen with higher accuracy.

With new developments and increased proficiency of recent times, more complex laparoscopic surgical techniques have been made clinically available. Exhibiting various superiorities, such as the requirement for only a single-incision, less trauma, less prominent cosmetic surgery scars, and convenient sample removal, SILS remains a developing technology [15–17] At present, this technique is extensively used in various surgical fields, such as gynecologic surgery [18], gastrointestinal surgery [17, 19], urological surgery [20], and hepatobiliary a surgery. Still, numerous studies have recently shown that single-incision laparoscopic surgery exhibits better cosmesis than open surgery, but it displays little in terms of other positive health benefits for aggressive treatment in patients [15, 21]. In addition, other studies have discovered that single-incision laparoscopic surgery bears no advantages over multi-incision laparoscopic surgery, as it increases the risk of surgical site infection and incision hernia [15, 22–24]. In 2000, Raboei et al. [25] presented the first case of adult SILS surgery. By contrast, SILS surgery in children was first reported in 2010 [26]. Raboei et al. [10] reported the application of SILS in a series of 39 children patients in 2019, the findings suggesting that this approach was both feasible and safe, and that it didn't increase postoperative complications. However, studies pertaining to massive splenomegaly, particularly in children, are inadequate. Since 2013, our hospital has carried out MILS surgery treatment for massive splenomegaly in children, obtaining satisfactory results. As such, our hospital initiated SILS surgery in 2018. In this research, to clarify the safety of SILS for massive splenomegaly in children, we discovered that no remarkable differences in statistics existed between the SILS group and MILS group in terms of bleeding volume, admission day, postoperative pain, and postoperative complications, which indicated that SILS was similar to MILS with respect to safety. However, the operative time for SILS proved longer than MILS. Still, a prolonged operation time can be accepted, considering the esthetic benefits of SILS surgery. Taken together, the single-incision laparoscopic method can decrease invasion while maintaining safeness and validity, optimizing surgery results, and improving cosmetic effects.

Operation time is an essential factor influencing the eventual outcome, as a longer operation duration may increase surgery-related complications [27, 28]. According to some research, operative time was remarkably longer in the laparoscopic group in contrast to the open group, whereas the time decreased significantly with the improvement in doctors’ proficiency. In other studies, the shorter operation duration in the laparoscopy group showed shorter times [29, 30]. Herein, the operation time for laparoscopic splenectomy was longer compared with open surgery, and the SILS group displayed a longer operation time relative to the MILS group, which was consistent with past research. By correlative analysis, we found that the operative time of MILS was primarily correlated with splenic length. Nevertheless, the operation time of SILS displayed a positive correlation with age, weight, height, and splenic length, which coincided with the outcomes of research from Targarona et al. [31]. The fact that the operation time of laparoscopic splenectomy increases with spleen size is consistent with the finding of Patel et al. [8]. The potential reasons are stated below: an enlarged spleen would reduce the volume of the abdominal cavity and increase the difficulty in removing surgical specimens, all of which would significantly prolong the operative time. Our team discovered that a child's height was positively and strongly associated with the operative time of SILS, which might be due to the disadvantages of single-incision laparoscopic surgery, including the mutual interference of surgical instruments, difficulty of surgical exposure, and poor surgical vision. To improve the view and decrease the mutual interference of surgical instruments, a 30° laparoscope and articulating or curved graspers or scissors were used by Budzyński et al. [32]. Traynor et al. [33] reported that operation time decreased with the increasing number of patients. Given improvements in single-incision laparoscopic techniques, SILS may be a secure substitution for multi-port laparoscopic splenectomy surgery in the future.

Additionally, available literature reported an overall 8–23.3% complication rate of laparoscopy splenectomy [34, 35], which included massive intraoperative bleeding, visceral injury, conversion to open surgery, surgical site infection, postoperative intra‐abdominal bleeding, intestinal obstruction, and systemic inflammation. Raboei et al. [10] confirmed that laparoscopic splenectomy surgery was safe and feasible, and when performed by a duly trained team, did not increase the risk of complications or the difficulty in surgery. To identify the independent factors promoting undesirable results in patients, the standards of adverse outcomes were ascertained via a questionnaire survey. The logistic regression analysis defined the independent risk factors for LS undesirable results, and this study confirmed that spleen size was an independent risk factor for undesirable results in LS. Notwithstanding, age, gender, BMI, height, weight, and surgery may not be independent risk factors for the adverse outcomes. This result further illustrated that the rate of SILS surgery complications in children was comparable to MILS surgery, even given a longer operative time, Shin et al. [36, 37] as spleen size was not related to the prevalence of post-operative complicating conditions. Overwhelming Post-Splenectomy Infection (OPSI) proves the most vital potential complicating disease posterior to splenectomy, the occurrence rate of which is 3–5%, and which frequently proves fatal [38, 39]. Five patients in this study presented OPSI, with two in the OS group and three in the LS group. All five patients achieved complete remission after valid anti-infection treatment. Portal Venous System Thrombosis (PVST) posterior to splenectomy is frequently an underlying cause of serious hepatic failure and mortality [35, 40]. The clinical presentation of PVST includes fever and abdominal pain, and if there are clinical manifestations, PVST can be accurately diagnosed by vascular ultrasound and CT [41, 42]. This study encountered one sufferer in the LS group that developed PVST. In contrast, sufferers in the OS group did not experience these complications. The treatment of PVST mainly includes anticoagulation and thrombolysis.

We acknowledge that this study bears several limitations. The present research is merely a retrospective study on the foundation of reliable data, and every patient was from a single-center. In addition, our team failed to perform external validation in additional datasets.

## Conclusion

Laparoscopic splenectomy is a secure and minimally invasive technique for treating massive splenomegaly in children. Our work suggests that either SILS surgery or MISL surgery can be considered a preferred regimen rather than merely an elective surgery for patients. OS surgery should be considered a better choice if patients cannot tolerate the prolonged operating time. Significantly, spleen size is the main contributor to the operative time and adverse outcomes of LS. Ultimately, SILS surgery for pediatric splenomegaly remains a technique in evolution, offering various cosmetic advantages, but presenting challenges both technically and logistically.
